# Hemophagocytic Lymphohistiocytosis Complicating Melioidosis

**DOI:** 10.4269/ajtmh.18-0331

**Published:** 2018-09

**Authors:** Simon Smith, Azhar Mohamed Munas, Josh Hanson

**Affiliations:** 1 Department of Medicine, Cairns Hospital, Cairns, Australia;; 2 James Cook University, Cairns, Australia;; 3 The Kirby Institute, New South Wales, Australia

**Figure 1.**
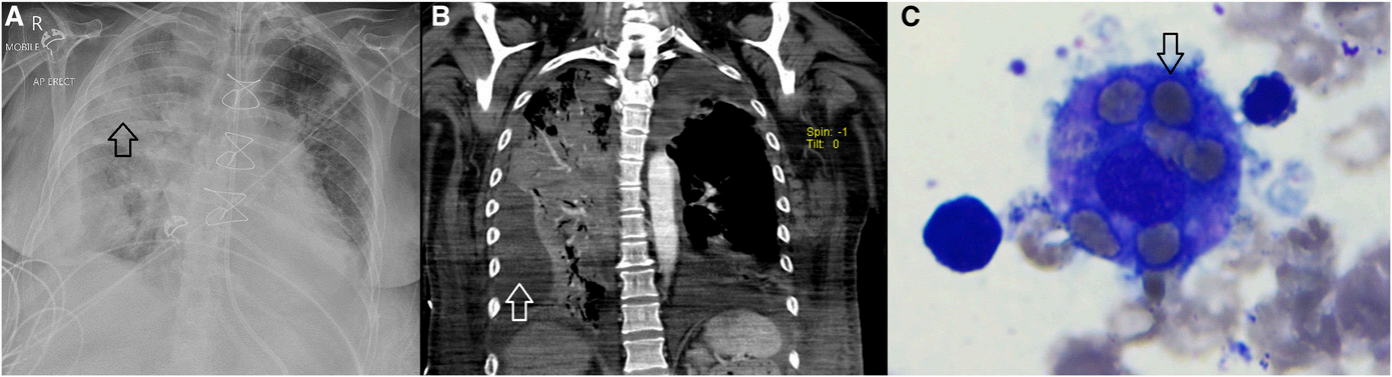
(**A**) Anteroposterior chest X-ray showing extensive consolidation (arrow). (**B**) Coronal computerized tomography images confirming extensive pneumonia and revealing a large right-sided pleural effusion (arrow). (**C**) A histiocyte with arrow pointing to mature erythrocyte phagocytosed within the cytoplasm. At least six mature erythrocytes are seen within this cell. This figure appears in color at www.ajtmh.org.

A 50-year-old Caucasian female living in remote northern Australia presented to hospital with a 2-day history of lower limb pain, back pain, and confusion. Her past medical history was notable for hazardous alcohol use and an aortic valve replacement 3 months previously. On arrival to hospital, her respiratory rate was 40 breaths per minute, heart rate was 114 beats per minute, and blood pressure was 137/67 mm of Hg. Her hemoglobin was 133 g/L, her leukocyte count was 7.0 × 10^9^/L, and her platelets were 211 × 10^9^/L. A chest X-ray revealed right upper lobe consolidation and she received empirical intravenous meropenem and azithromycin. She rapidly deteriorated and required intubation for respiratory failure. Blood cultures collected on admission subsequently grew *Burkholderia pseudomallei*, confirming a diagnosis of melioidosis. She had a tumultuous course in the intensive care unit; on day one, she required vasopressor support to maintain adequate tissue perfusion, and on day 3, she developed thrombocytopenia (platelets 72 × 10^9^/L) and anemia (hemoglobin 113 g/L). A repeat chest X-ray and computerized tomography scan of the lungs revealed extensive consolidation and a loculated right-sided pleural effusion which required thoracentesis (Figure 1). Despite continued meropenem, she failed to improve, and by day 21, she had developed marked pancytopenia (hemoglobin 68 g/L, leukocytes 1.2 × 10^9^/L, and platelets 14 × 10^9^/L). Her neutropenia did not improve despite granulocyte colony-stimulating factor and a bone marrow aspirate revealed hemophagocytosis (Figure 1). She was persistently febrile, her serum ferritin was 1,220 μg/L, and fibrinogen was 0.9 g/L, consistent with a diagnosis of hemophagocytic lymphohistiocytosis (HLH). She continued to deteriorate despite ongoing antibiotic therapy and died on day 41 of her admission.

Secondary HLH can occur in association with a number of conditions, including infections, malignancy, immunodeficiency, and rheumatological diseases. The diagnosis can be established by molecular testing or by fulfillment of specified clinical and laboratory criteria.^1^ Hemophagocytic lymphohistiocytosis is associated with a poor prognosis, with mortality rates greater than 40%.^2^ Viruses are more likely to trigger HLH than bacteria; however, a number of bacterial infections have been implicated^2^; melioidosis should be added to the list. Melioidosis, a disease endemic in northern Australia,^3^ is commonly associated with cytopenias,^4,5^ particularly in the setting of trimethoprim/sulfamethoxazole therapy. However, if cytopenias are severe or persistent, an alternative pathology should be considered.
